# Report on the Types and Distribution of Antennal Sensilla in Lygaeidae (Heteroptera: Lygaeoidea) and Their Putative Functions

**DOI:** 10.3390/insects16010044

**Published:** 2025-01-06

**Authors:** Agnieszka Nowińska

**Affiliations:** Faculty of Natural Science, Institute of Biology, Biotechnology and Environmental Protection, University of Silesia in Katowice, Bankowa 9, 40-007 Katowice, Poland; agnieszka.nowinska@us.edu.pl

**Keywords:** sensilla, morphology, Lygaeidae, antennae

## Abstract

Antennal sensillar structures respond to a variety of environmental stimuli. In the present study, the antennal sensilla of all three subfamilies of the family Lygaeidae are studied for the first time. This research shows the presence of five main types of sensilla (sensilla trichodea, chaetica, campaniformia, basiconica, and coeloconica). Their comparison to other, previously studied, heteropteran insects and their putative mechano-, chemo-, and thermo-hygroreceptive functions are discussed. The current study shows uniformity in the types of sensilla in all studied species. However, minor differences between the subfamilies, as well as within the subfamily Lygaeinae, are observed.

## Simple Summary

Insects can receive environmental signals via structures called sensilla. Different types of sensilla receive different kinds of stimuli. In this paper, the antennal sensilla of three subfamilies of seed bugs (Lygaeidae) are analyzed to outline potential similarities and differences between them, as well as to discuss their potential function.

## 1. Introduction

Insects perceive the environment through the use of receptive organs called sensilla. 

They are structures of different shapes and sizes (they are also commonly called sensory hairs or sensory pegs) that receive different types of signals from the environment [1,2,3]. While an array of different sensilla are present on a range of insect body parts, the antennae are believed to play a crucial role in their life, as they receive stimuli connected to food and nest locations, the suitability of environmental conditions, mating, and inter- and intra-specific recognition [4,5].

The external morphology of the sensilla is easily accessible for study. Therefore, many detailed studies were conducted on the shapes of these structures (hairlike, peglike, platelike, etc.), as well as on their surfaces (smooth or with the presence of pores) and types of connection with the cuticle (flexible and inflexible sockets). These studies were later extended with the support of ultrastructural studies of sensilla, which revealed different numbers of neurons, dendrites, which were branched or not branched, as well as the presence of sheath cells. Ultrastructural studies allowed for the assignment of functions to sensilla displaying specific external morphologies [6,7,8,9,10]. Presently, scientists are able to hypothesize, to some degree, on the function of sensilla based on their external morphology.

The family Lygaeidae is a taxon belonging to the superfamily Lygaeoidea (Insecta: Hemiptera: Heteroptera). They are commonly known as seed bugs, since they are primarily seed feeders. They are also called ground bugs or milkweed bugs, as some of the species also feed on sap. They are found on every continent except Antarctica [11]. A few species, especially of the genus *Spilostethus*, are recorded as pests on agricultural crops [12,13,14]. The species of the family vary in size and form. Representatives of the subfamilies Ischnorhynchinae and Orsillinae are smaller and of bleak coloration. On the other hand, members of the subfamily Lygaeinae are bigger and often bright red or yellow and black [15].

The taxon was introduced in 1829 by Samuel Peter Schilling [16]. The definition of Lygaeidae used to be much wider and included the representatives of modern families such as Artheneidae, Blissidae, Cryptorhamphidae, Cymidae, Geocoridae, Heterogastridae, Ninidae, Oxycarenidae, and Ryparochromidae. However, in 1997, Thomas J. Henry introduced the most recent classification, where the aforementioned taxa, former subfamilies, were removed from Lygaeidae and given an independent family status [17]. Presently, the family Lygaeidae comprises three subfamilies: Ischnorhynchinae, Lygaeinae, and Orsilinae [18].

In the order Heteroptera, the antennal sensilla of an array of taxa were already extensively studied. Research was conducted on many representatives of aquatic and semi-aquatic bugs—Nepomorpha and Gerromorpha [19,20,21,22,23,24]—as well as the terrestrial Reduviidae and Pentatomidae [25,26,27,28,29,30,31,32,33,34]. Some data are also available on Aradidae, Miridae, or Coreidae [35,36,37,38,39]. These structures remain to be studied in the majority of the superfamily Lygaeoidea. Research was conducted only on the last antennal segments of *Elasmolomus pallens* (Dallas, 1852) (Ryparochromidae) and *Oncopeltus fasciatus* (Dallas, 1852) (Lygaeidae) [40,41].

The present study aims to investigate the antennal sensilla of all three subfamilies of Lygaeidae for the first time. The morphology of different types is investigated and compared with other studied lygaeid and heteropteran insects and their possible functions are discussed.

## 2. Materials and Methods

### 2.1. Taxon Samples

Materials in the form of dry specimens were borrowed from the collections of the Moravian Museum in Brno, Czech Republic, and the Naturalis Biodiversity Center in Leiden, the Netherlands. Both sexes were examined, in 2–5 specimens of each species. Both males and females were studied. However, no notable differences in the types and distribution of sensilla between the sexes were observed. Therefore, the sex of the specimen was not taken into consideration in the presentation of the results.

The antennae of 11 species from all three subfamilies of Lygaeidae were studied.
Ischnorhynchinae:
*Kleidocerys resedae* (Panzer, 1793)Lygeinae
*Apterola pedestris focarilei* Tamanini, 1964

*Cosmopleurus fluvipes* (Dallas, 1852)

*Graptostethus servus* (Fabricius, 1787)

*Lygaeus equestris* (Linnaeus, 1758)

*Lygaeus oreophilus* (Kiritshenko, 1931)

*Lygaeus saxatilis* Scopoli, 1763

*Spilostethus hospes* (Fabricius, 1794)Orsilinae:Nysiini*Nithecus jacobaeae* (Schilling, 1829)

*Nysius thymi* (Wolff, 1804)
Orsillini*Orsillus depressus* (Mulsant & Rey, 1852)

### 2.2. Preparation and Imaging

The antennae of dry specimens were dissected and cleaned with the use of an ultrasonic cleaner (Polsonic, Warsaw, Poland). The samples were then dried in a series of ethanol solutions (30%, 50%, 70%, 80%, 86%, and 90% for 30 min and 100% for 1 day) and mounted onto aluminum pin stubs. To prepare them for imaging, they were sputtered with a 30 nm layer of gold with the use of a turbomolecular pump coater (Quorum 150 T ES plus—Quorum Technologies, Laughton, East Sussex, UK).

After the preparation, the samples were analyzed with the use of scanning electron microscopes in the scanning microscopy laboratory of the Faculty of Natural Science, Institute of Biology, Biotechnology and Environmental Protection of the Silesian University in Katowice (Katowice, Poland): a Phenom XL (Phenom-World BV, Eindhoven, The Netherlands) and a Hitachi UHR FE-SEM SU8010 (High Technologies, Tokyo, Japan).

Additionally, the measurements of the lengths of the antennae of 42 specimens were taken and the method of standard deviation was applied.

Figures and schematics were made and edited with the use of Adobe Photoshop, CorelDRAW Graphic Suite CC 2015, and Paint.net 5.1.2.

The terminology for sensilla and their putative functions based on their morphologies were adapted from Altner & Prilinger [7], Zacharuk [42], and Shields [10].

## 3. Results

### 3.1. General Morphology of the Antennae of Lygaeidae

**Figure 1:**
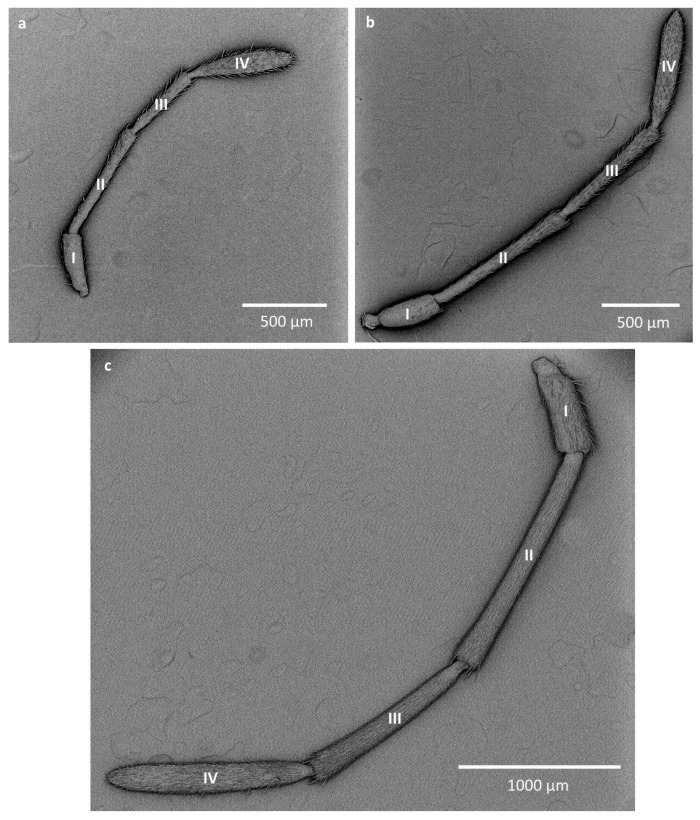
Antennae of representatives of three studied families: (**a**) *Kleidocerys resedae* (Ischnorhynchinae), (**b**) *Orsillus depressus* (Orsillinae), (**c**) *Cosmopleurus fluvipes* (Lygeinae); I—scapus; II—pedicel; III—basiflagelloreme; IV—distiflagellomere.

The antennae of Lygaeidae are uniform in shape among the studied representatives. They consist of four antennomeres. The first antennomere (scapus) is the shortest and is much wider than the next two. The second (pedicel) and third (basiflagellomere) antennomeres are thin and long, with the second antennomere being generally longer than the third and, in the majority of the studied species, more or less double the length of the first antennomere. The fourth antennomere (distiflagellomere) is thin at the base, widening in the middle part to narrow slightly at the end of the antennomere (Figure 1, Table 1).

### 3.2. Types of Sensilla Observed on the Antennae of Lygaeidae

In total, five main types of sensilla were observed on the antennae of the studied lygaeid species: sensilla trichodea (ST), sensilla chaetica (SCh), sensilla campaniformia (SCa), sensilla basiconica (SB), and sensilla coeloconica (SCo). Their distribution on the antennae in each studied species is presented in Table 2.

**Figure 2:**
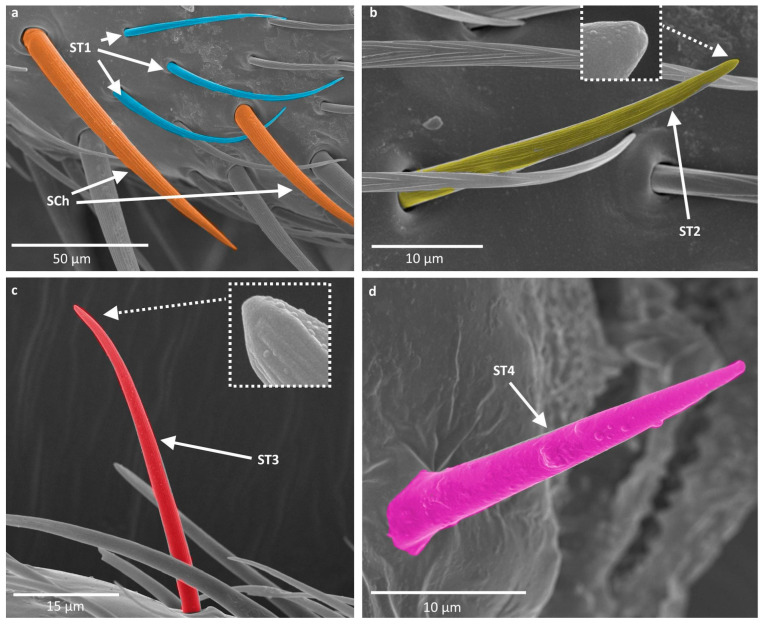
(**a**–**d**) types of sensilla observed on the antennae of studied species; ST—sensilla trichodea; SCh—sensilla chaetica. The colors correspond to the ones used throughout the article.

Sensilla trichodea (ST) are generally long, hairlike structures with a flexible socket, which means they possess a thin membrane that connects the cuticle of the antennae with the cuticle of the sensillum, allowing its movement at the base. This characteristic allows for the assignment of their putative function—mechanoreception. Their surface is usually ribbed and lacks pores and the tip of the sensillum can be sharp or blunt. In the studied species, they constitute the majority of sensilla found on the antennae and are distributed on all antennomeres. A few subtypes of this main type were observed:

ST1 are straight and long sensilla, tapering at the tip, with well pronounced ribs. They constitute the majority of sensilla present in the studied species and were observed on all antennomeres. They differ in width and length. The biggest difference can be observed at the end of the pedicel and basiflagellomere, where the sensilla are much longer and wider and they resemble sensilla chaetica (Figure 2a).

ST2 are sensilla shorter than ST1, with less pronounced ribs and a slightly blunt tip. They do not grow towards the tip of the antenna, like most other ST, but visibly protrude from its surface. At the tip of sensillum the ribs appear to be fading away and a pore at the tip of sensillum appears to be present. These characters might imply a gustatory function of the sensillum. To confirm or deny this statement, however, ultrastructural studies need to be conducted. This subtype generally appears on the pedicel and basiflagellomere (in some species, singular small ST2 were also observed near the end of the first antennomere) and the sensilla are sparsely distributed along the length of the antennomere (Figure 2b).

ST3 are long sensilla with slightly pronounced ribs. They appear to be longer than other sensilla trichodea and protrude from the surface of the antenna. They are only present on the distiflagellomere, along its length, and at the tip. They were examined for the presence of a pore at the tip but, because of their movement resulting from electron bombardment, they were difficult to observe. The achieved results do not confirm or deny the presence of a pore at the tip. Ultrastructural studies need to be performed in order to confirm or deny its presence (Figure 2c).

ST4—also described as Böhm bristles—are cone-shaped sensilla with no pores or ribs on the surface. Their tips are usually blunt, and they are observed on the base of the scapus and pedicel. This type of sensillum is believed to perform a proprioceptive function (Figure 2d).

Sensilla chaetica (SCh) are long, straight structures, generally thicker than sensilla trichodea. They are embedded in a flexible socket; therefore, they are believed to play a mechanoreceptive role. Their surface possesses well-developed ribs and lacks pores, and their tips can be blunt or tapered. They were observed, in general, on the scapus, where they occur among sensilla trichodea (Figure 2a).

**Figure 3:**
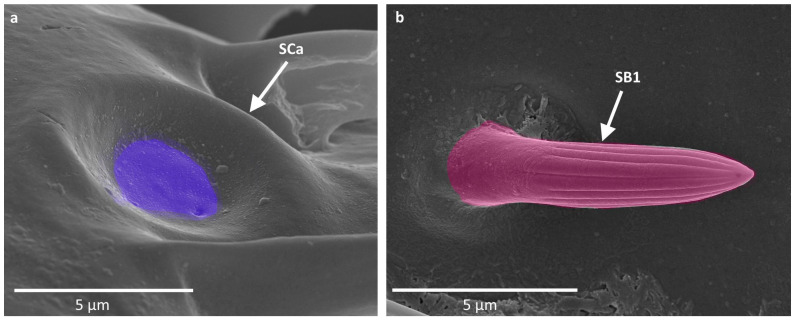
(**a**,**b**) types of sensilla observed on the antennae of studied species; SCa—sensilla campaniformia; SB—sensilla basiconica. The colors correspond to the ones used throughout the article.

Sensilla campaniformia (SCa) are flat and round or elongated disk structures with a smooth surface, a molting pore in the middle, and a flexible socket. They are believed to be mechanoreceptors that respond to pressure. In the studied species, they were observed in groups on the base of the scapus and singularly along the length of the antennae (Figure 3a).

Sensilla basiconica (SB) are structures of different lengths embedded in an inflexible socket (no additional membrane between the cuticle of the antenna and the cuticle of the sensillum). In general, they have a porous surface, often with subtle or well visible ribs. The presence of pores implicates their putative chemoreceptive (olfactory) function. In the studied species, they were observed only on the distiflagellomere. Three subtypes of this main type were described:

SB1 are short, cone-like structures, positioned either on the flat surface or embedded in a small cavity. They have a smooth base whereas the rest of the sensillum is ribbed. This type of sensilla generally occurs in insects and possesses pores in between the ribs. They were observed on the distiflagellomere, occurring singularly or in groups (Figure 3b).

**Figure 4:**
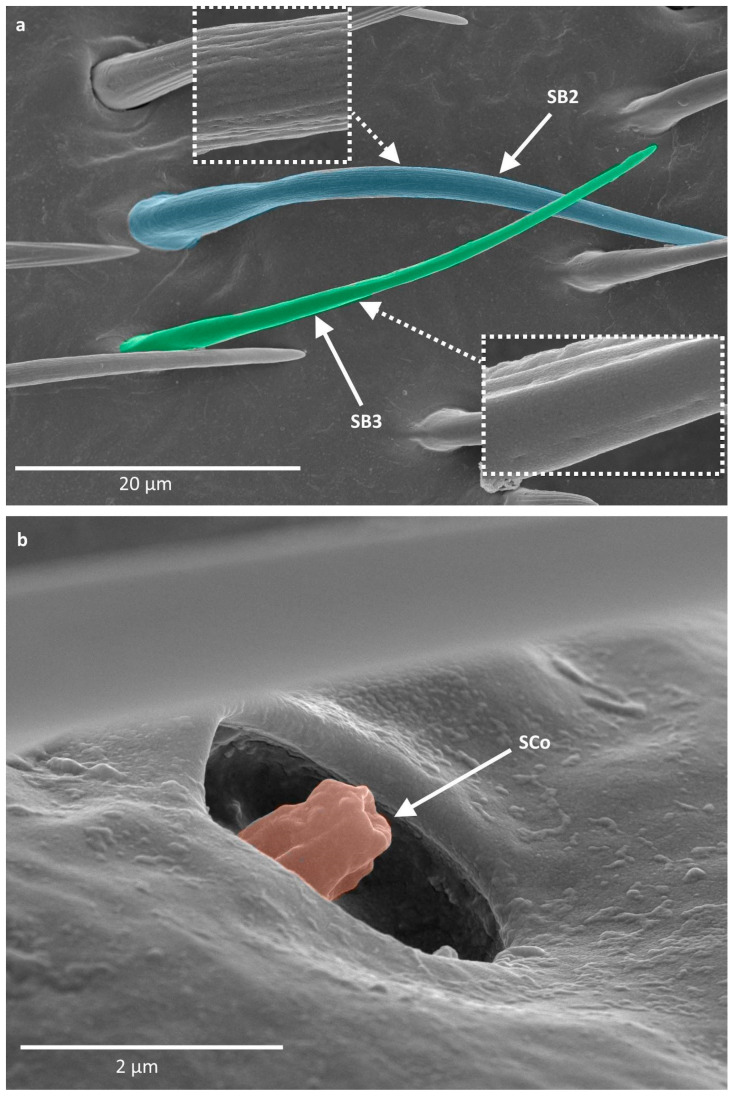
(**a**,**b**) types of sensilla observed on the antennae of studied species; SB—sensilla basiconica; SCo—sensilla coeloconica. The colors correspond to the ones used throughout the article.

SB2 are long structures with a blunt tip, smooth at the base. The rest of the body of the sensillum possesses ribs. They either have a round cross-section along the whole length or are flattened in some part of their body. They are generally present in the middle part of the distiflagellomere (Figure 4a).

SB3 are long and thin structures with a smooth base and a few singular ribs present at the distal part. They possess pores, although they are not as easily visible as in SB2. In the studied species, SB3 occurred along SB2 on the majority of the surface of the distiflagellomere (Figure 4a).

Sensilla coeloconica (SCo) are small structures with an inflexible socket, embedded in a cavity. They have a smooth or slightly ribbed surface with an opening at the tip. In the studied species, they were observed on the distiflagellomere and also at the end of the pedicel and basiflagellomere (Figure 4b).

**Figure 5:**
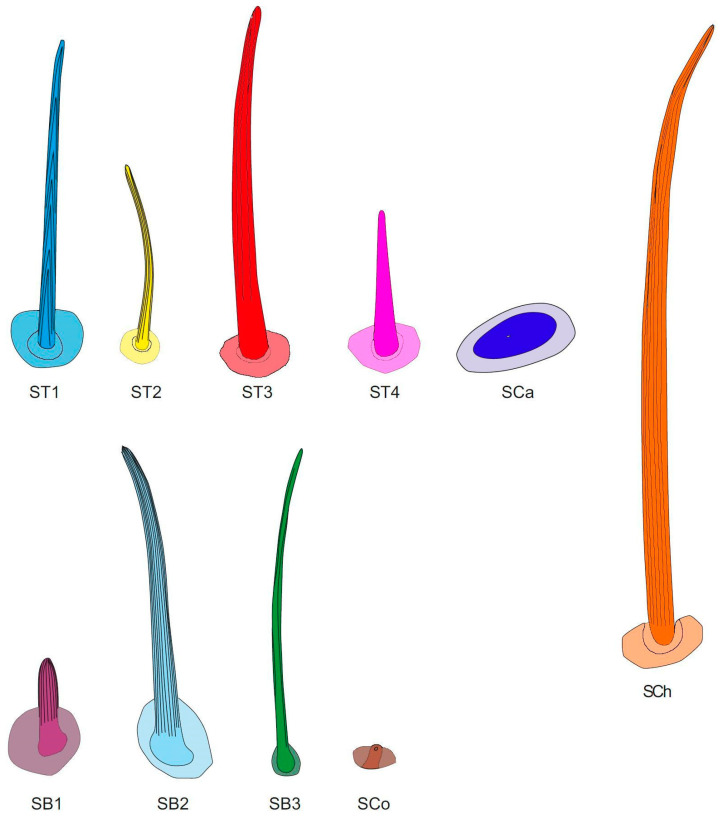
Schematics of the types of sensilla observed in the studied species. ST—sensilla trichodea; SCa—sensilla campaniformia; SCh—sensilla chaetica; SB—sensilla basiconica; SCo—sensilla coeloconica. The colors correspond to the ones used throughout the article.

The schematics of the described types of sensilla are presented in Figure 5.

### 3.3. Distribution of the Sensilla on the Antennae of Lygaeidae

**Figure 6:**
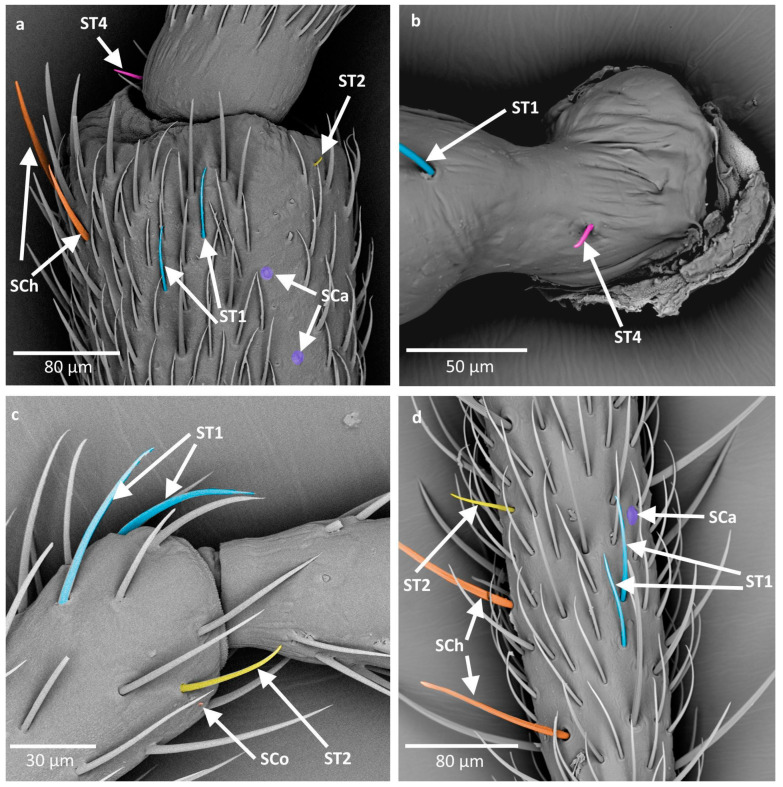
Distribution of sensilla in studied species: (**a**) scapus of *C. fluvipes*, (**b**) scapus of *N. jacobaeae*, (**c**) pedicel of *K. resedae*, (**d**) pedicel of *A. pedestris*. ST—sensilla trichodea; SCh—sensilla chaetica; SCa—sensilla campaniformia; SCo—sensilla coeloconica. The colors correspond to the ones used throughout the article.

**Figure 7:**
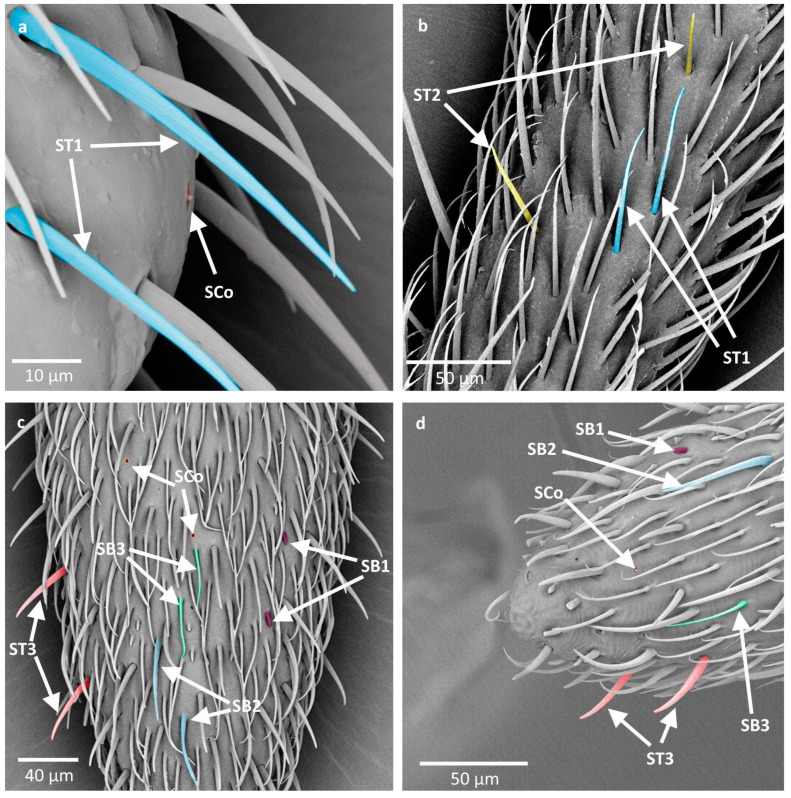
Distribution of the sensilla in the studied species. (**a**) The end of the basiflagellomere of *O. depressus*, (**b**) basiflagellomere of *G. servus*, (**c**) distiflagellomere of *L. saxatilis*, (**d**) distiflagellomere of *S. hospes*. ST—sensilla trichodea; SCo—sensilla coeloconica; SB—sensilla basiconica. The colors correspond to the ones used throughout the article.

The general pattern of distribution was similar in all studied species. On the scapus, ST4 and SCa were observed at the base, with SCa present along the length of the antennomere. The majority of the antennomere was covered with ST1 and SCh, with a singular ST2 present near the end of the antennomere in most of the studied species. SCh were not observed only in *N. jacobaeae*, *N. thymi,* and *O. depressus*. The pedicel was also generally covered with ST1, with ST2 in smaller numbers appearing among them. On the base of the segment, ST4 was observed in almost all the studied specimens. In the majority of the studied species (with the exception of *A. pedestris*, *S. hospes*, *N. thymi,* and *O. depressus*), one or two SCo were observed at the end of the antennomere. SCa scattered along the length of the antennomere were also present in the majority of studied species. In *A. pedestris* and *G. servus*, SCh were present among the other sensilla trichodea (Figure 6). The basiflagellomere generally displayed the same distribution pattern as the pedicel. A lack of SCo at the end of the segment was observed in *G. servus* and *N. thymi*. Again in *A. pedestris* and *G. servus*, SCh were present among the other sensilla trichodea. In *A. pedestris*, *G. servus,* and *N. thymi*, ST4 were observed on the base of the basiflagellomere. ST1 were present on the base part of the distiflagellomere. ST3, SB1, SB2, and SB3 were observed on the middle and distal parts. ST3, SB2, and SB3 displayed a distribution pattern similar to sensilla trichodea’s pattern on the other antennomeres. They covered the antennomere at regular intervals (Figure 7). SCa were observed scattered along the length of the antennomere and SCo were singularly present on the surface of the segment. However, in most of the studied species, singular SCo were observed at the end of the antennomere.

**Figure 8:**
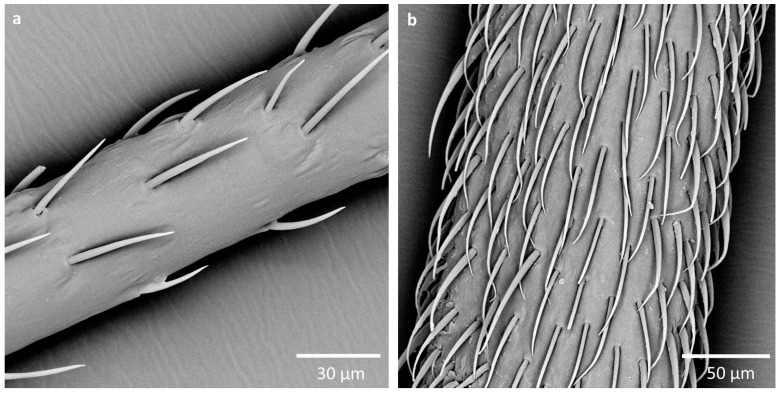
Distribution of sensilla trichodea on the pedicel: (**a**) *N. thymi*, (**b**) *L. equestris*.

Apart from the distribution of the different types of sensilla on the antennae, a difference in the distribution of sensilla trichodea (especially on the pedicel and basiflagellomere) was visible. In most of the studied species, sensilla trichodea densely covered the surface of the antennomere. However, in *K. resedae*, *N. jacobaeae*, *N. thymi*, and *O. depressus*, these sensilla were much more sparsely but very regularly distributed (Figure 8).

## 4. Discussion

The present study is the first thorough study on the morphology of the antennal sensilla in representatives of all three subfamilies of the family Lygaeidae.

### 4.1. The Sensory Equipment of the Antennae of Lygaeidae

#### 4.1.1. Mechanoreception

Mechanoreceptive sensilla in insects usually act as tactile receptors which detect the movement of objects in the environment. Mechanical stimuli are involved in behavioral activities like locomotion, posture, feeding, and orientation, thus making mechanoreceptors very important structures in insects’ bodies [2,9].

The ultrastructures of antennal mechanoreceptors, especially trichoid and chaetic sensilla, were investigated in hemipteran species [43,44,45] as well as other taxa [46,47,48], the results of which support the putative function assigned based on the morphologies of the presently studied sensilla.

Three main types of sensilla with a putative mechanoreceptive function were observed in the studied species—sensilla trichodea, sensilla chaetica and sensilla campaniformia. In all studied species, sensilla trichodea ST1 covered the majority of the surface of the first three antennomeres, proving that they are important structures for the sensory apparatus.

Sensilla chaetica (SCh), as well as long and thick sensilla trichodea, were observed in the studied species of Lygaeinae and Ischnorhynchinae. Sensilla chaetica were mostly present on the scapus, which is the one that often has a lot of contact with the head. Long and thick sensilla trichodea, on the other hand, were observed at the end of the pedicel and basiflagellomere, which are the areas of connection between the antennomeres. The size and position of these sensilla indicate that they might be important for contact mechanoreception.

Two types of sensilla trichodea with a possible contact chemoreceptive function (ST2 and ST3) were observed in all the studied species. ST2 were present on the scapus, pedicel, and basiflagellomere, whereas ST3 were observed on the distiflagellomere. Contact chemoreceptors can be recognized by possessing a blunt tip which is perforated by a single pore [2]. The position of the observed structures and the indication of a pore at the tip might suggest a putative contact chemoreceptive function. However, the observation of a tip pore on long, hairlike sensilla with the use of the scanning electron microscope is extremely difficult due to their constant movement. Therefore, ultrastructural studies need to be conducted in order to confirm or deny this suspicion for this specific sensillum.

Sensilla trichodea ST4 were observed at the base of the scapus in all studied species and, in the majority of them, on the base of the pedicel. In *A. pedestris*, *G. servus* and *N. thymi*, a singular ST4 was observed on the base of the basiflagellomere. The lack of ribs and the position of the sensilla between the antennomeres clearly indicate their proprioceptive function.

Sensilla campaniformia SCa were also observed in all the studied specimens. They are grouped at the base of the scapus but also scattered along the surfaces of all the other antennal segments. Their putative function is responding to stresses in the exoskeleton [2].

The high number of mechanoreceptive sensilla found on the antennae of Lygaeidae clearly proves their importance to these insects. Since their antennae are long and constantly moving, all types of mechanical stimuli need to be processed.

These types of sensilla are also commonly found in hemipteran species [19,20,21,22,23,24,25,26,27,28,29,30,31,32,33,34,35,36,37,38,39]

#### 4.1.2. Chemoreception

Chemoreceptive olfactory organs perceive airborne chemicals due to the presence of multiple pores on the surface. Chemoreception is crucial for environmental recognition, e.g., food or mating partner location [43].

The ultrastructure of chemoreceptors, in the form of basiconic sensilla in insects, was already extensively studied [3,4,6,7,10,43,44,45,46,47,48]. Moreover, the function of olfactory sensilla was proven by the recognition of odorant binding proteins in different insect species [49,50].

Three types of sensilla basiconica with a putative olfactory function were observed in the present study. Short sensilla SB1 were observed in clusters or singularly scattered. Long sensilla SB2 and SB3 were distributed regularly, in a pattern resembling the pattern of distribution of the aforementioned trichoid sensilla ST1. They cover most of the surface and occur in large numbers. All types of sensilla basiconica, in all of the studied species, were uniform in form and distribution and were found only on the distiflagellomere.

These findings are confirmed by other studies on the antennal sensilla of terrestrial, semi-aquatic and aquatic insects, which found chemoreceptive structures to be present mostly on the most distal antennal segment [19,23,35].

#### 4.1.3. Thermo-Hygroreception

Heat and moisture sensors in the form of blunt pegs or cones, with a terminal molting pore and enclosed inside a pit, are often found in insects [7,9,51].

The ultrastructure of thermo-hygroreceptors, especially sensilla coeloconica, was of interest to many scientists [7,43,44,45,46,47,52,53]. Extensive research was conducted in ant species, including extracellular recordings of neuronal activity, in order to understand and support their function [54].

In the present study, sensilla coeloconica SCo were found in all of the studied species. In general, their numbers were small and, in some species, only singular structures were found. They were observed on the distiflagellomere, either along the surface and/or near the tip of the antennomere. In many studied species, they were also present at the end of the pedicel and/or basiflagellomere. Their small size and rare occurrence (especially near the end of the antennomeres) makes it difficult to spot them on the antennae. Therefore, not all of their possible structures might have been discovered.

Sensilla coeloconica are important to insects living in any environment, and therefore a great number of these structures was found among the heteropteran taxa [19,23,35].

### 4.2. The Diversity of Types and Distribution of Antennal Sensilla Among the Studied Subfamilies

The main difference observed between the studied subfamilies is without a doubt the distribution of sensilla trichodea on the pedicel and basiflagellomere. In species from the subfamilies Ischnorhynchinae and Orsilinae, sensilla trichodea are sparsely distributed on the antennomeres, while in representatives of Lygaeinae, the number and density of these sensilla are much higher. While this difference is evident and allows for a clear distinction between the subfamilies, the sizes of the antennae should also be taken into consideration. The body, and therefore the antennae of Lygaeinae are much greater in size [15]. A bigger size (and therefore antennal surface area) allows for more sensilla to be present on the antennae, thus it is possible that no other factors influence this result.

The size of the antennae might also influence the presence of bigger antennal sensilla. It might explain the lack of sensilla chaetica in the species of the subfamily Orsilinae. Sensilla chaetica are present on the scapus of *K. resedae* but in much smaller numbers than in the studied representatives of Lygaeinae.

Another factor that slightly differentiates the antennae of the studied species is the presence of SCa and SCo on different antennomeres. However, due to their small size, these structures are not easy to observe with the use of the scanning electron microscope and probably should not be taken into consideration as a clear differentiating factor.

In general, except for minor differences in types and distribution of the antennal sensilla, the sensilla patterns on the antennae of all studied subfamilies of Lygaeidae are very uniform. To draw any strong conclusions, especially on the antennal sensilla significance in the family’s systematic position, a wider study on the antennae of other lygaeid taxa is needed.

### 4.3. Comparison of Antennal Sensilla of Lygaeidae with Other Studied Lygaeoidea and Heteroptera

One species from the family Lygaeidae (*Oncopeltus fasciatus*) was studied by Harbach and Larsen [41], with the use of scanning and transmission electron microscopes. The authors described sensilla on the distal region of the distiflagellomere. In their results, they presented four main types of sensilla: sensilla basiconica, sensilla trichodea, sensilla coeloconica, and sensilla chaetica. The described structures were observed in the present study on other Lygaeidae. However, two of the types are classified differently in the present study. Sensilla chaetica described in *O. fasciatus* are classified as sensilla trichodea ST4 in the present study. The sensilla described as trichodea in *O. fasciatus* were described as sensilla basiconica SB3 in the present study due to the observed inflexible socket and the presence of pores on its surface. Harbach and Larsen also presented the ultrastructure of this type of sensillum, which, judging by the presence of multiple dendrites, supports the hypothesis that the sensillum is olfactory. In the photograph presenting their results on the ultrastructure of sensilla chaetica (in this study described as ST4), they did not observe apparent dendrites and hypothesized on the presence of a pore tubule. Since the result is inconclusive, the function of this type of sensillum cannot yet be proven with certainty.

Another study on *Elasmolomus sordidus*, from the family Ryparochromidae, was carried out by Rani and Madhavendra [40]. It provides even fewer data on the types of sensilla observed on the distiflagellomere. A few subtypes of sensilla trichodea and basiconica with sharp and blunt tips were described and, based on the description and figure presented in the study, they correspond to sensilla basiconica SB1, SB2, SB3, and trichodea ST4 in the present study.

None of the studies, however, shows the presence of sensilla campaniformia SCa, which, in the present study, was observed even on the distiflagellomere.

In comparison to other, previously studied Heteroptera, all the main sensilla types found in Lygaeidae (trichodea, chaetica, campaniformia, basiconica, and coeloconica) correspond to the types present in other studied taxa [25,33,34,36].

The sensillar set of water bugs is much wider in variety and possesses more types of putative mechanoreceptive sensilla, in comparison to the presently studied species, which might be connected to their environment [23]. While Lygaeidae do not present a lot of diversity in putative mechanoreceptive structures, mechanoreceptors constitute the majority of their antennal sensilla, reinforcing their importance.

In studies on other terrestrial Heteroptera, it has been suggested that sensilla trichodea could also be involved in contact chemoreception [31,32]. In the present study, this function is also hypothesized for two types of sensilla trichodea.

Sensilla that were not observed in Lygaeidae are sensilla placodea with a putative olfactory function. They were reported in semi-aquatic bugs and in one pentatomid species [19,34]. This type was also reported in many other terrestrial insects’ taxa [55,56,57,58,59].

## 5. Conclusions

Antennal sensilla responsible for the detection of different stimuli—with putative mechano-, chemo-, and thermo-hygroreceptive functions—were observed in three subfamilies of Lygaeidae. Sensilla trichodea, chaetica, campaniformia, basiconica, and coeloconica of different subtypes were present. In total, nine different morphological types were described, six of them representing putative mechanoreceptive structures. Some minor differences between the studied subfamilies were observed, as well as small variations among the species of Lygaeinae. However, the general sensillar set and the distribution of different types on the antennae of Lygaeidae display a uniformity of these structures among the studied taxon.

## Figures and Tables

**Table 1:** Size of antennomeres in the studied species.

Species Name	Length of the Scapus (µm)	Length of the Pedicel (µm)	Length of the Basiflagellomere (µm)	Length of the Distiflagellomere (µm)
*Kleidocerys resedae*	390 ± 23.77	714 ± 60.30	507 ± 25.68	637 ± 19.66
*Apterola pedestris focarilei*	642 ± 51.39	1225 ± 40.59	907 ± 104.51	1022 ± 101.43
*Cosmopleurus fluvipes*	691 ± 45.57	1524 ± 70.00	1173 ± 58.19	1273 ± 17.39
*Graptostethus servus*	713.5 ± 15.80	1482 ± 61.47	1108 ± 24.75	1408 ± 34.22
*Lygaeus equestris*	744 ± 96.55	1659 ± 36.77	1282 ± 26.95	1605 ± 46.37
*Lygaeus oreophilus*	680 ± 23.11	1431 ± 30.41	1161 ± 67.18	1467 ± 20.51
*Lygaeus saxatilis*	895 ± 71.74	1740 ± 87.16	1256 ± 60.44	1696 ± 89.68
*Spilostethus hospes*	698 ± 37.14	1630 ± 54.50	1496 ± 16.80	1667 ± 16.80
*Nithecus jacobaeae*	517 ± 50.82	857 ± 66.26	694 ± 59.17	773 ± 12.30
*Nysius thymi*	426 ± 45.77	800 ± 22.48	579 ± 21.47	635 ± 14.86
*Orsillus depressus*	504 ± 37.05	1052 ± 46.67	866 ± 20.51	823 ± 27.58

**Table 2:** Distribution of the types of sensilla in the studied species. The colors correspond to the ones used throughout the article.

Species Name	Sensilla on the Scapus	Sensilla on the Pedicel	Sensilla on the Basiflagellomere	Sensilla on the Distiflagellomere
*Kleidocerys resedae*	ST1, ST4 SCh SCa	ST1, ST2, ST4 SCa SCo	ST1, ST2 SCa SCo	ST1, ST3 SCa SB1, SB2, SB3 SCo
*Apterola pedestris*	ST1, ST2, ST4 SCh SCa	ST1, ST2, ST4 SCh SCa	ST1, ST2, ST4 SCh SCa SCo	ST1, ST3 SCa SB1, SB2, SB3 SCo
*Cosmopleurus fluvipes*	ST1, ST2, ST4 SCh SCa	ST1, ST2, ST4 SCa SCo	ST1, ST2 SCa SCo	ST1, ST3 SCa SB1, SB2, SB3 SCo
*Graptostethus servus*	ST1, ST2, ST4 SCh SCa	ST1, ST2, ST4 SCh SCa SCo	ST1, ST2, ST4 SCh	ST1, ST3 SCa SB1, SB2, SB3 SCo
*Lygaeus equestris*	ST1, ST4 SCh SCa	ST1, ST2 SCa SCo	ST1, ST2 SCo	ST1, ST3 SCa SB1, SB2, SB3 SCo
*Lygaeus oreophilus*	ST1, ST2, ST4 SCh SCa	ST1, ST2, ST4 SCa SCo	ST1, ST2 SCa SCo	ST1, ST3 SCa SB1, SB2, SB3 SCo
*Lygaeus saxatilis*	ST1, ST2, ST4 SCh SCa	ST1, ST2, ST4 SCa SCo	ST1, ST2 SCa SCo	ST1, ST3 SCa SB1, SB2, SB3 SCo
*Spilostethus hospes*	ST1, ST4 SCh SCa	ST1, ST2, ST4 SCa	ST1, ST2 SCo	ST1, ST3 SCa SB1, SB2, SB3 SCo
*Nithecus jacobaeae*	ST1, ST2, ST4 SCa	ST1, ST2 SCa SCo	ST1, ST2 SCa SCo	ST1, ST3 SCa SB1, SB2, SB3 SCo
*Nysius thymi*	ST1, ST4 SCa	ST1, ST2, ST4 SCa	ST1, ST2, ST4	ST1, ST3 SCa SB1, SB2, SB3 SCo
*Orsillus depressus*	ST1, ST2, ST4 SCa	ST1, ST2, ST4	ST1, ST2 SCa SCo	ST1, ST3 SB1, SB2, SB3 SCo

## Data Availability

The data that support the findings of this study are available from the corresponding author, [A.N.], upon reasonable request.
